# Synthesis of Tyrosol and Hydroxytyrosol Glycofuranosides and Their Biochemical and Biological Activities in Cell-Free and Cellular Assays

**DOI:** 10.3390/molecules26247607

**Published:** 2021-12-15

**Authors:** Peter Kis, Eva Horváthová, Eliška Gálová, Andrea Ševčovičová, Veronika Antalová, Elena Karnišová Potocká, Vladimír Mastihuba, Mária Mastihubová

**Affiliations:** 1 Institute of Chemistry, Slovak Academy of Sciences, 845 38 Bratislava, Slovakia; peter.kis@savba.sk (P.K.); elena.potocka@savba.sk (E.K.P.); vladimir.mastihuba@savba.sk (V.M.); 2 Cancer Research Institute, Biomedical Research Center, Slovak Academy of Sciences, 845 05 Bratislava, Slovakia; eva.horvathova@savba.sk; 3 Department of Genetics, Faculty of Natural Sciences, Comenius University, 842 15 Bratislava, Slovakia; eliska.galova@uniba.sk (E.G.); andrea.sevcovicova@uniba.sk (A.Š.); veron.antalova@gmail.com (V.A.)

**Keywords:** tyrosol, hydroxytyrosol, phenylethanoid glycofuranosides, basic zinc carbonate, antioxidant activity, cytotoxicity, DNA damage, DNA-protective effect

## Abstract

Tyrosol (T) and hydroxytyrosol (HOT) and their glycosides are promising candidates for applications in functional food products or in complementary therapy. A series of phenylethanoid glycofuranosides (PEGFs) were synthesized to compare some of their biochemical and biological activities with T and HOT. The optimization of glycosylation promoted by environmentally benign basic zinc carbonate was performed to prepare HOT α-L-arabino-, β-D-apio-, and β-D-ribofuranosides. T and HOT β-D-fructofuranosides, prepared by enzymatic transfructosylation of T and HOT, were also included in the comparative study. The antioxidant capacity and DNA-protective potential of T, HOT, and PEGFs on plasmid DNA were determined using cell-free assays. The DNA-damaging potential of the studied compounds for human hepatoma HepG2 cells and their DNA-protective potential on HepG2 cells against hydrogen peroxide were evaluated using the comet assay. Experiments revealed a spectrum of different activities of the studied compounds. HOT and HOT β-D-fructofuranoside appear to be the best-performing scavengers and protectants of plasmid DNA and HepG2 cells. T and T β-D-fructofuranoside display almost zero or low scavenging/antioxidant activity and protective effects on plasmid DNA or HepG2 cells. The results imply that especially HOT β-D-fructofuranoside and β-D-apiofuranoside could be considered as prospective molecules for the subsequent design of supplements with potential in food and health protection.

## 1. Introduction

**Figure 1:**
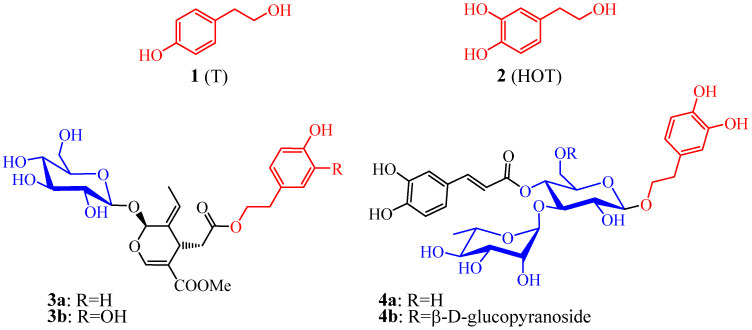
Representatives of phenylethanoids isolated from olive tree by-products or medicinal plants.

Epidemiological studies indicate that plant-derived foods exert some beneficial effects on human health [1], particularly on age-related diseases [2]. Phenylethanoid antioxidants tyrosol (**1**, T) and hydroxytyrosol (**2**, HOT) (Figure 1) are relatively broadly spread in nature and are intensively studied for such properties [3,4]. They constitute structural parts of natural compounds such as ligustroside (**3a**), oleuropein (**3b**), and phenylethanoid glycosides (PEGs) [5,6,7,8,9]. While the sources of **3a** and **3b** are agroindustrial by-products from olive mills [10], PEGs have been found in various plant-based foods, such as edible flowers and tea [8]. Moreover, PEG acteoside (also known as verbascoside, **4a**) has been found in olive tree by-products, alongside T, HOT, and **3a** and **3b** [10,11] (Figure 1). Olive metabolites **2**, **3b**, and **4a** appear to act as topoisomerase II poisons in complex preparations intended for human consumption [12].

Nowadays, there is growing scientific interest focused on HOT, which is a promising candidate for application in functional food products or in complementary therapy. HOT is by far the most investigated olive polyphenol, possibly not only due to its antioxidant power [13] and tasteless character, but also because it is a product of oleuropein **3a** degradation. It can be found in high concentrations in olive oil mill wastewater, produced during mechanical olive processing within olive oil production [14]. A wide variety of health-beneficial effects attributable especially to HOT and their potential therapeutic applications were recently reviewed [15,16,17]. Further reviews focused on mapping its high antioxidant capacity and important anti-inflammatory, anticancer, cardioprotective, and neuroprotective effects [18,19,20]. HOT was recently approved as a novel safe food additive [21], and there is an increasing number of papers on its large-scale isolation from natural resources [14] or chemical and biotechnological production [18,22,23] due to the interest of the food, feed, supplementary, and pharmaceutical sectors.

The unsubstituted HOT is, however, rather unstable and has other shortcomings. For example, the rapid metabolism of HOT leads to the low bioavailability of the active substance and the bioavailability depends to a large extent on the vehicle in which HOT is administered [24,25,26]. HOT was found to be a potent OH^•^, O_2_^•^^−^, and ONOOH scavenger but a poor scavenger of HOCl and H_2_O_2_ [13]. Tailored functionalization of natural phenols can upgrade their properties [27,28]. The chemical modification of HOT can help to improve their characteristics, such as chemical stability, solubility, and bioavailability, or to increase the antioxidant or biological activity by altering the pharmacokinetic profile. Natural diphenols are often used as scaffolds to prepare new, effective biologically active drugs. There have been developed synthetic strategies to broaden the applications of HOT in foodstuffs, cosmetics, and pharmaceuticals [29,30]. 

One of the possibilities to extend the applications of HOT is glycosylation of its primary OH group without impacting the catechol moiety in order to preserve the antioxidant properties. The use of carbohydrates as scaffolds or pharmacophores in the field of polyphenolic compounds can result in a variety of structures with different spatial arrangements, reactivity to enzymes, or the ability to bind to cellular receptors. Structurally, this type of glycophenolic occurs in a large group of natural bioactive substances: PEGs. PEGs are water-soluble compounds widely distributed in medicinal plants. They are characterized by a hydroxyphenylethyl moiety (mostly HOT) attached to β-*D*-glucopyranose (rarely β-*D*-allopyranose) through 1,2-*trans* glycosidic linkage. The central glucopyranoside may be decorated by a hydroxyphenylpropenoyl moiety and/or other monosaccharides [5,6,7,8,9]. Acteoside **4a** and echinacoside **4b** (which is a constituent of various nutritional supplements from *Echinacea purpurea*), depicted in Figure 1, are some of the best-known representatives of PEGs. 

Chemical preparation of T or HOT glycosides usually requires several steps of synthesis. In general, T or HOT protected on the phenolic groups is glycosylated by a protected activated glycosyl donor and the product is ultimately deprotected [31,32]. The weak point of such synthesis may be the insufficient stereoselectivity of the glycosylation and the eventual purification of the mixture of anomers [33]. The controlled regioselective attachment of additional monosaccharides and/or a phenylpropanoid moiety to the molecules requires the application of a sophisticated synthetic strategy [34,35]. Direct glycosylation of T or HOT is possible by a selective enzyme transglycosylation of suitable glycosyl donor [35,36,37,38,39,40,41,42,43]. Some glycosidases, however, do not distinguish between the phenolic hydroxyl and the primary hydroxyl of phenylethanoid acceptors [44,45]. 

Glycofuranosides [46,47] are widely spread in nature, oftentimes in the form of oligo- or polysaccharides. While D-fructofuranosides [48], L-arabinofuranosides [49], and D-apiofuranosides [50] are found in plants, D-galactofuranosides and D-arabinofuranosides are present in microorganisms [51]. In addition, D-ribofuranosides and 2-deoxy-D-ribofuranosides are essential in nucleic acids. Glycofuranosyl-containing conjugates may have different biological or physicochemical properties than analogous glycopyranosides. They are recognized in plants and microorganisms by different enzymes [52,53,54,55,56]. Non-digestible plant fructooligosaccharides and arabinoxylans have prebiotic effects [57].

To the best of our knowledge, T or HOT glycofuranosides have not yet been isolated from nature, but the existence of other natural furanosyl-containing polyphenols is known. For example, myricetin-3-*O*-α-L-arabinofuranoside isolated from *Calycolpus warszewiczianus* displays weak antimalarial activity [58] or (–)-catechin-7-*O*-β-D-apiofuranoside from *Ulmus davidiana var. japonica* inhibits hepatic stellate cell activation [59]. As a result, structurally well-defined synthetic furanosyl analogs of PEGs are of great interest for their potential therapeutic applications.

Our laboratory has experience in the preparation of phenolic arylalkyl glycosides by conventional chemical synthesis [32,33,60] as well as enzymatic transglycosylation [38,39,40,61]. As a part of our research program directed toward PEGs, we have recently synthesized a series of natural and unnatural phenylethanoid glycopyranosides (PEGPs). Then, we evaluated their antioxidant properties (reducing power, DPPH radical scavenging, and Fe^2+^-chelating activity) as well as DNA-protective potential using cell-free approaches as well as an experimental system using in vitro cultured human cells. The PEGPs cytotoxicity on human hepatoma HepG2 cells (MTT test) and the protective potential against lesions induced by a model DNA-damaging agent (H_2_O_2_; comet assay) were assessed. While hydroxysalidroside proved to be the best candidate in cell-free experiments, salidroside was effective in protection at the cellular level at all tested concentrations [32].

In this work, we intended to prepare HOT glycofuranosides (α-L-arabino-, β-D-apio-, and β-D-ribofuranoside) as analogues to HOT glycopyranosides and to investigate their potential in the same biochemical and biological in vitro experiments. At the same time, we wanted to compare the potential of aglycones (T, HOT) as well as previously enzymatically prepared β-D-fructofuranosides [39].

## 2. Results and Discussion

### 2.1. Synthesis of Hydroxytyrosol Glycofuranosides

**Figure 2:**
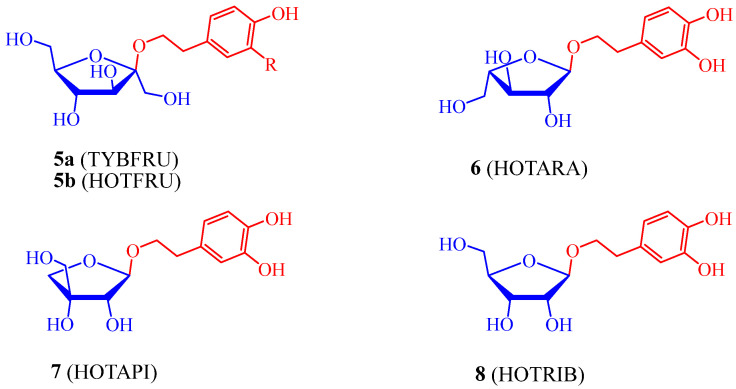
Overview of prepared and tested PEGFs.

T and HOT β-D-fructofuranosides **5a** and **5b** (TYBFRU and HOTFRU) (Figure 2) were prepared from sucrose by enzymatic transfructosylation catalyzed by yeast β-galactosidase Lactozym 3000 L comprising invertase activity [39]. Generally, the chemical synthesis of β-fructofuranosides is difficult due to the *cis*-position of the OH group on C-3 with the aglycone on C-2 in β-D-fructofuranosides. The possibility of efficient preparation of **5a** and **5b** with invertase with high synthetic activity was, therefore, an advantage. When structurally different glycosides are to be prepared, enzymatic glycosylation is complicated by the need of appropriate enzyme with suitable substrate specificity. Recently, the possibility of enzymatic preparation of β-D-apiosides (3-*C* branched D-erythrofuranosides) was investigated in our laboratory, but no enzyme with a transapiosylating activity was found, since all tested apiosidases were able to catalyze only apioside hydrolysis [62,63]. We, therefore, decided to study suitable conditions for chemically promoted 1,2-*trans*-glycofuranosylation and to prepare HOT α-L-arabinofuranoside (**6**, HOTARA), HOT β-D-apiofuranoside (**7**, HOTAPI), and HOT β-D-ribofuranoside (**8**, HOTRIB) in this way (Figure 2).

We decided to build on our previous work [33], in which we studied environmentally friendly methods of the modified Koenigs–Knorr glycosylation using zinc oxide (ZnO) or basic zinc carbonate ([ZnCO_3_]_2_·[Zn(OH)_2_]_3_) as a promoter. These inexpensive and environmentally benign Zn(II) promoters have been found to be efficient and selective for 1,2-*trans-*glucosylation of tyrosol and hydroxytyrosol with the phenolic hydroxyls protected by easily removable acetyl or *t*-butyldimethylsilyl groups. Various per-*O*-acetylated hexopyranosyl, pentopyranosyl, and rungiosyl bromides have been successfully used to determine the viability and scope of this method [33]. For this study, we chose a more stereoselective glycosylation method promoted by basic zinc carbonate. Similarly to the synthesis of analogous pyranosides, the standard procedure for preparing 1,2-*trans*-furanosides uses glycosyl donors that have acyl protecting groups at O-2 [64,65].

**Scheme 1:**
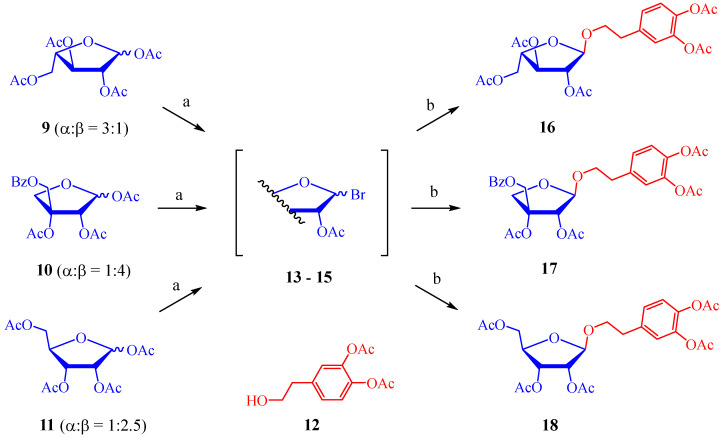
Glycofuranosylation of protected HOT **12**. Reagents and conditions: (**a**) TMSBr (5.0 equiv), CH_2_Cl_2_, 4 °C—rt, 4–15 h, **13**–**15** quant %; (**b**) **12** (0.8 equiv), basic ZnCO_3_, toluene, MS 4 Å, 60 °C, yields of **16**–**18** in Table 1.

To ensure the furanose form of the products **6**–**8**, it was important to prepare starting per-*O*-acylated furanoses **9**–**11** (Scheme 1). 1,2,3,5-Tetra-*O*-acetyl-α,β-L-arabinofuranose (**9)** was prepared in a three-step synthesis according to Backinowski et al. from L-arabinose [66]. The pentofuranose **9** was obtained as an anomeric mixture with anomer composition α:β = 3:1. 5-*O*-Benzoyl-1,2,3-tri-*O*-acetyl-α,β-D-apiofuranose (**10**) (α:β = 1:4) was obtained from 2,3-*O*-isopropylidene-α,β-D-apiofuranose by enzymatic benzoylation, acid-catalyzed deisopropylidenation, and acetylation [62]. The starting 2,3-isopropylidenated D-apiofuranose was obtained from D-mannose in several steps [67,68]. Per-*O*-acetyl-α,β-D-ribofuranose **11** (α:β = 1:2.5) was synthesized in a similar manner to the synthesis of peracetyl-D-arabinofuranose **9** by the Guthrie–Smith method [69]. The kinetic product methyl D-ribofuranoside obtained by acidic methanolysis was acetylated, followed by acetolysis of the methyl group. Aglycone **12** (HOT protected on phenolic moieties by acetylation) was prepared from the corresponding hydroxyphenylacetic acid by a two-step sequence: protection of phenolic groups and reduction of the carboxyl group [33].

Furanosyl bromides 2,3,5-tri-*O*-acetyl-L-arabinofuranosyl bromide **13**, 2,3,5-tri-*O*-acetyl-D-ribofuranosyl bromide **14**, and 2,3-di-*O*-acetyl-5-*O*-benzoyl-D-apiofuranosyl bromide **15** as activated glycosyl donors were prepared in one step and in high yields starting from the corresponding peracylated furanoses **9**–**11**. A solution of trimethylsilyl bromide in CH_2_Cl_2_ was used for quantitative bromination [70]. Due to the high instability of the bromides, the reaction mixtures were carefully concentrated, so that the temperature did not exceed 40 °C, and used directly for further glycosylations.

Basic ZnCO_3_ (0.35–0.44 equiv) was used as a promoter in toluene to optimize glycofuranosylation. The reaction of glycosyl donors **13**–**15** (1.25 equiv) with acceptor **12** (1 equiv) was conducted with addition of 4 Å MS to prevent the formation of hydrolysis products. The reaction was carried out at 60 °C under conventional heating as well as under microwave irradiation (Table 1). Glycosylation of **12** by pentofuranosyl bromides **13**–**15** proceeded with higher stereoselectivity than the reaction with conformationally different pyranosyls studied in our previous work [33], giving only 1,2-*trans*-furanosides under conventional heating at 60 °C (Table 1, entries 1, 3, and 6). The reactivity under the studied conditions was comparable to the reactivity of reactive pentopyranosyls and higher than the reactivity of hexopyranosyls [33]. By using basic zinc carbonate as a promoter upon microwave irradiation, the reaction times were similarly significantly reduced from hours to minutes but the stereoselectivity of products had declined (Table 1, entries 2, 3, and 5) and some amount of 1,2-*cis*-furanosides was observed in ^1^H NMR spectra. We applied the best glycosylation conditions (entries 1, 3, and 6) to the reactions in preparative scale, and furanosides **16**–**18** were isolated in yields of about 70%. 

**Scheme 2:**
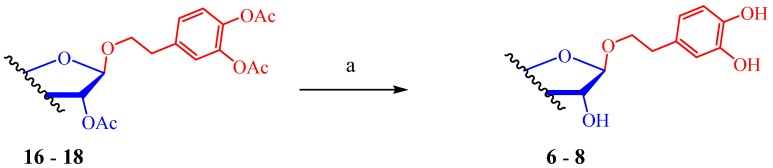
Deprotection of furanosides. Reagents and conditions: (**a**) 0.5 M MeONa/MeOH, 4 °C—rt, **6** 44%, **7** 36%, and **8** 31%.

In the final synthetic step, the removal of the acetyl groups (benzoyl group for apiofuranozid **17**) under Zemplén conditions proceeded smoothly (Scheme 2) but purification of the target glycosides **6**–**8** was laborious. The phenolic substances were absorbed and probably oxidized on silica gel, and the yields were, therefore, lower (30–40%). Trouble-free purification of polyphenols by preparative chromatography still remains a challenge.

### 2.2. Cell-Free Assays

#### 2.2.1. Reducing Power Assay and DPPH Radical Scavenging Activity

The electron-donating ability (Fe^3+^/Fe^2+^ reduction) of the studied PEGFs **5a**, **5b**, and **6**–**8** was used to monitor their antioxidant properties. The reducing power of gallic acid (GA) was compared with all other tested compounds. Based on experimental results, we can conclude that HOT and hydroxytyrosol analogues (HOTFRU, HOTARA, HOTAPI, and HOTRIB) exhibited the most potent and concentration-dependent reduction capacity (Table 2). The reducing effect of T and TYBFRU in the concentration range tested was much lower than that of GA as well as the other hydroxytyrosol analogues **5b** and **6**–**8** (Table 2).

The DPPH assay was used to compare the radical scavenging activity of the tested compounds. As shown in Table 2, the DPPH radical scavenging of HOT and HOT furanosides (HOTFRU, HOTARA, HOTAPI, and HOTRIB) was comparable with GA (known to be a strong antioxidant agent and used as a standard). T and TYBFRU exhibited the lowest DPPH-radical-scavenging activities (Table 2).

The highest reducing power and antioxidant activity demonstrated by HOT and HOT furanosides detected by us and as reviewed also by Karković et al. [3] could be attributed to the catechol moiety in their structures. 

#### 2.2.2. Assessment of DNA-Damaging/-Protective Potential

**Figure 3:**
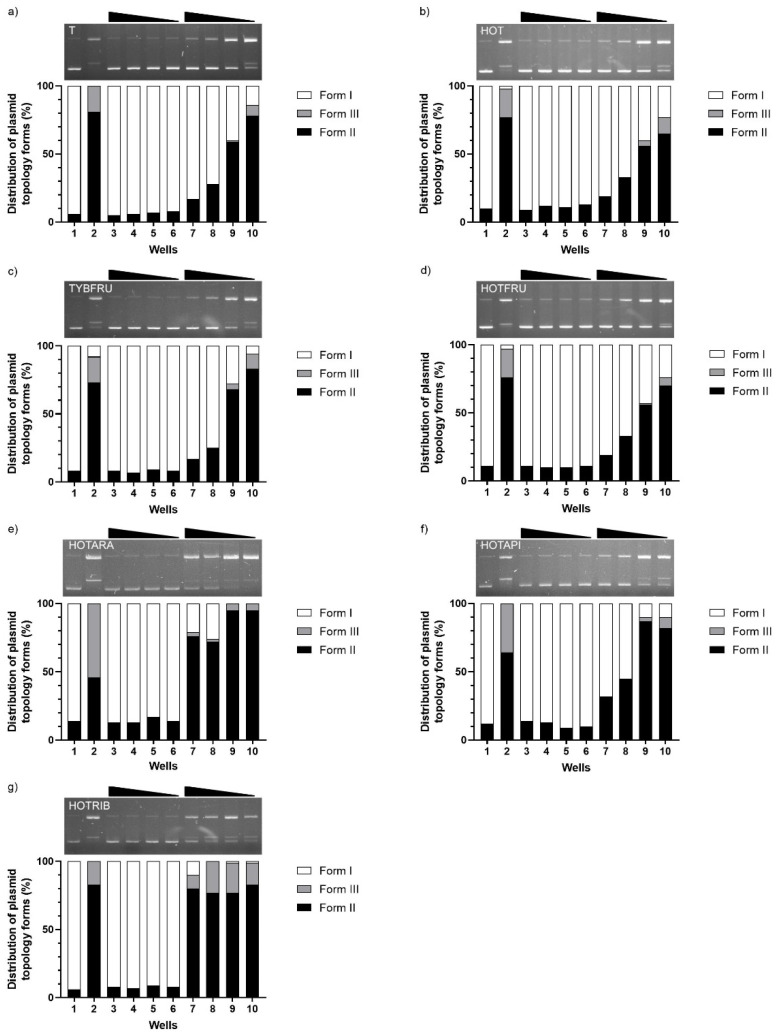
DNA-protective potential (DNA topology assay) of aglycones (**a**) T (**1**) and (**b**) HOT (**2**) and PEGFs (**c**) TYBFRU (**5a**), (**d**) HOTFRU (**5b**), (**e**) HOTARA (**6**), (**f**) HOTAPI (**7**), and (**g**) HOTRIB (**8**) against Fe^2+^-induced DNA damage in plasmid pBR322 presented as electrophoretograms as well as densitometric distribution of plasmid topology forms (%). The order of wells in each gel: 1, NS or negative standard (intact pBR322); 2, PS or positive standard (pBR322 treated with 10 µM Fe^2+^); 3–6, pBR322 treated with decreasing concentrations of T, HOT, and PEGFs (10–0.01 mM); 7–10, pBR322 treated with decreasing concentrations of T, HOT, and PEGFs (10–0.01 mM) in the presence of Fe^2+^ ions. Fe^2+^ ions induce DNA breaks via a Fenton-like reaction, resulting in the conversion of plasmid topology from supercoiled (form I) to relaxed circular (form II) and/or linear (form III).

Electrophoretic monitoring of structural changes induced in plasmid DNA by treatment with the tested compounds is shown in Figure 3. Analogues were tested for their DNA-damaging effects (pBR322 plasmid DNA treated with different concentrations of tested compounds; wells 3–6) and their potential DNA-protective effects in the presence of Fe^2+^ ions (wells 7–10). Fe^2+^ treatment of pBR322 plasmid generated single-strand and double-strand breaks, resulting in plasmid relaxation into open circular (form II) or linear (form III) forms (Figure 3, well 2). Similarly to the negative standard represented by the supercoiled pDNA (form I) (Figure 3, well 1), none of the tested compounds changed the mobility of the supercoiled pDNA topoisomers in the given concentration ranges (0.01–10 mM). Moreover, DNA-protective effects of T, HOT, TYBFRU, HOTFRU, and HOTAPI were manifested in a dose-dependent manner (Figure 3a–d,f). HOTARA and HOTRIB displayed only weak DNA-protective activities, as bands representing open relaxed circular and linear forms were present at all tested concentrations (Figure 3e,g). 

The phenylethanoids (T and HOT) are assumed to be folded into the *gauche* conformation of the hydroxyethyl chain, in which its OH group is oriented toward the aromatic ring [32]. This *gauche* conformer is stabilized by the presence of the OH···π intramolecular interaction. A similar geometry of phenylethanoid aglycone was also suggested for glycopyranosides. Five-membered rings of glycofuranoses may adopt the envelope or twist conformations. According to observed vicinal interaction constants (^3^*J*) from ^1^H NMR spectra of studied HOT glycofuranosides, we can assume the conformations of their furanoside rings to be from ^3^T_2_ to ^1^E. Protons H-1 and H-2 of pentofuranosides **6**–**8** are pseudoequatorial and protons H-3 and H-4 of fructofuranoside **5a** are pseudoaxial (*J*_1,2_ (**6**) = 1.7 Hz; *J*_1,2_ (**7**) = 2.4 Hz; *J*_1,2_ (**8**) = 3.7 Hz; *J*_2,3_ (**6**) = 3.7 Hz; *J*_2,3_ (**8**) = 4.7 H; *J*_3,4_ (**5b**) = 8.1 Hz [39]). These considerations are also generally consistent with a review article published by Lowary et al. [47].

**Scheme 3:**
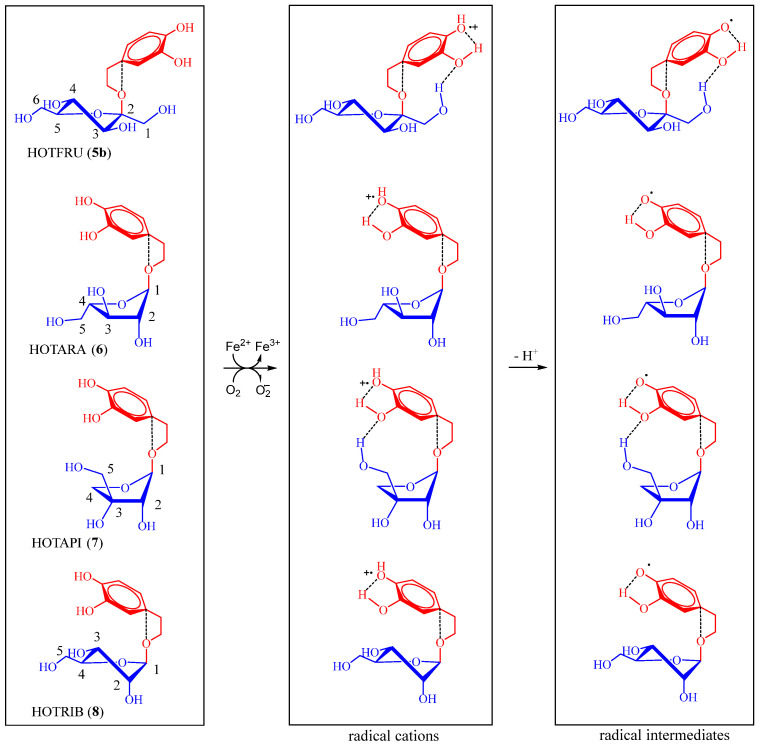
Probable conformers of **5b** and **6**–**8** and a plausible scavenging mechanism through a two-step sequential proton loss electron transfer reaction.

When comparing the results of the DNA topology assay for T and HOT or TYBFRU and HOTFRU, the compounds containing two *ortho* phenolic groups in their molecule were evidently more potent in the protective effect against DNA damage induced by Fe^2+^. Larger differences in the protective efficacy are perhaps visible between the individual HOT glycofuranosides HOTFRU and HOTAPI compared to HOTARA and HOTRIB (Figure 3). This supports our theory that if the HOT glycoside can acquire such a conformation that the free primary group of the CH_2_OH monosaccharide ring is close to the catechol moiety of the aglycone, it can help to stabilize the generated radical by forming a hydrogen bond [32]. Such a molecule geometry is excluded for HOTARA (**6**). The CH_2_OH group and aglycone are on the opposite sides of the plane of the furanoside ring. *Ortho*-diphenols of HOTFRU aglycone could interact in the oxidation state with CH_2_OH on the C-1 furanoside ring. The primary CH_2_OH group on the C-3 furanoside ring (HOTAPI, **7**) is likely to be more favorable for interaction than the one on C-4 (HOTRIB, **8**) (Scheme 3).

The potential interaction of the appropriate hydroxyl groups of glycofuranosides (such as primary 6-OH along with secondary 4-OH in HOTFRU) with the phosphate groups of plasmid DNA through the formation of a hydrogen bond could also affect the protective potential of a particular glycoside [32].

### 2.3. Cellular Assays

#### 2.3.1. Cell Viability

**Figure 4:**
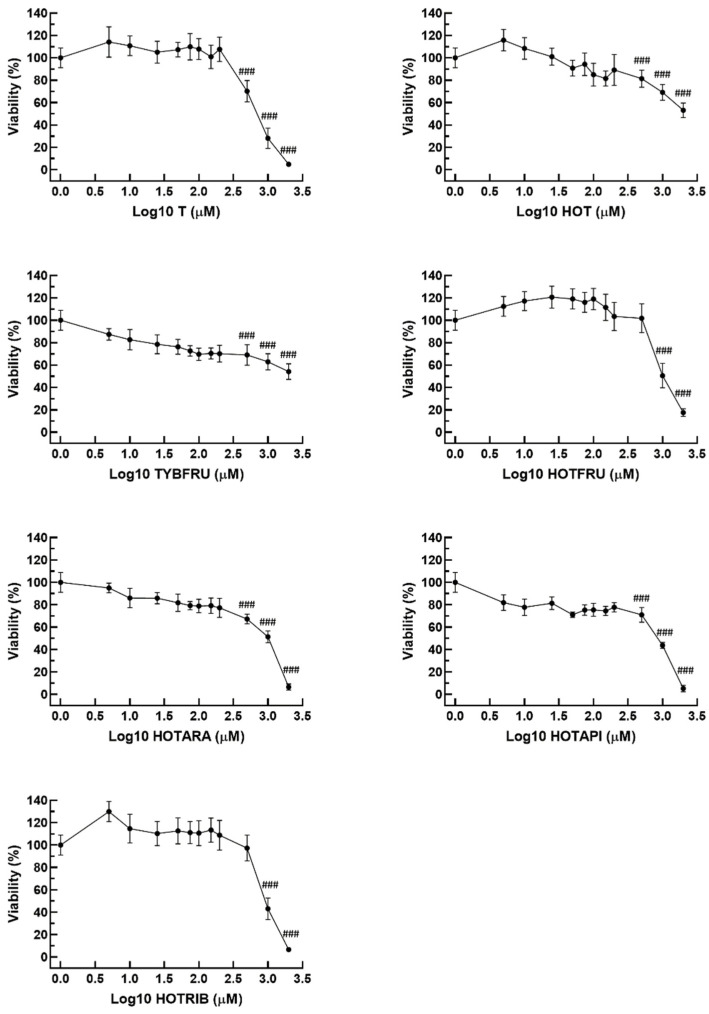
Viability of human hepatoma HepG2 cells (MTT test) after 48 h treatment with T, HOT, and PEGFs **5a**, **5b**, and **6**–**8**; ### *p* < 0.001 significant results in comparison to the negative standard (HepG2 cells treated with the solvent DMSO).

The cytotoxicity of T, HOT, and synthesized PEGFs on HepG2 cells was evaluated by the MTT assay, a sensitive method for detecting the cellular toxicity via the measurement of enzymatic conversion of MTT in the mitochondria [71,72]. Loss of cell viability was significant after a 48 h exposure of HepG2 cells to T, HOT, TYBFRU, HOTARA, and HOTAPI at the three highest concentrations (500–2000 μM), whereas for HOTFRU and HOTRIB, it was significant even at the two highest concentrations used for the treatment (1000 and 2000 μM), respectively. At 5–200 μM concentrations, T, HOT, and PEGFs did not reduce the viability of HepG2 cells (Figure 4). 

#### 2.3.2. Evaluation of Potential Protective Effects on HepG2 Cells

**Figure 5:**
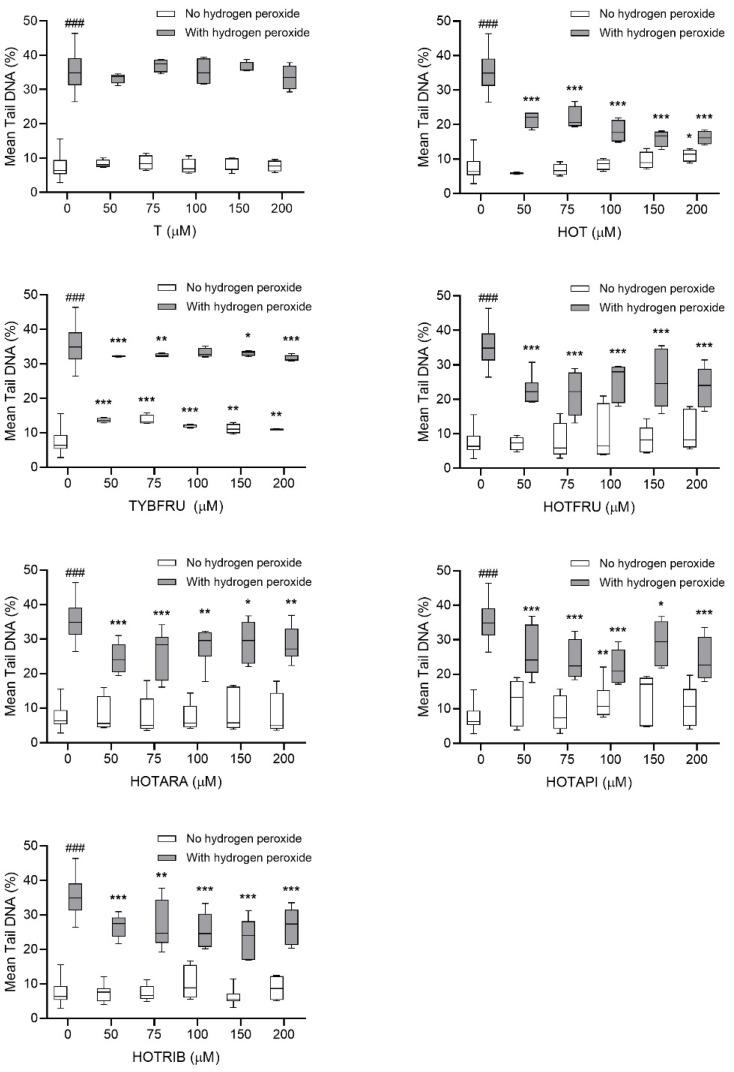
DNA damage (comet assay) caused by the combined treatment of T, HOT, and PEGFs **5a**, **5b**, and **6**–**8** (48 h) and H_2_O_2_ (300 μM, 5 min on ice) in human hepatoma HepG2 cells; ### *p* < 0.001 significant results in comparison to the negative standard (HepG2 cells treated with the solvent DMSO); * *p* < 0.05, ** *p* < 0.01, and *** *p* < 0.001 significant reduction in DNA damage in comparison to the positive standard (HepG2 cells treated with H_2_O_2_).

The potential DNA-damaging/-protective effects of T, HOT, and PEGFs at the concentrations ranging from 50 to 200 μM were investigated using the comet assay on human hepatoma HepG2 cells. The conventional alkaline comet assay showed a slight increase in DNA damage after TYBFRU treatment at all concentrations tested, after 200 μM HOT treatment, and after 100 μM HOTAPI treatment (Figure 5).

The protective activity of T, HOT, and PEGFs was assessed from the decrease in DNA damage induced by H_2_O_2_ alone. Experimental results showed that 48 h pre-treatment of HepG2 cells with HOT, hydroxytyrosol PEGFs, and TYBFRU before exposure to H_2_O_2_ for 5 min caused a significant decrease in DNA migration in the comet tails in all tested concentrations compared to the positive standard (Figure 5). In comparison with H_2_O_2_ alone (35.55%), which was used as a positive standard, it can be concluded that all the tested compounds except tyrosol (T) exert protective effects on human hepatoma HepG2 cells. Although TYBFRU exhibited a slight DNA-damaging effect in comparison with the negative standard, it manifested a significant DNA-protective effect when applied as a pre-treatment, unlike T, which neither induced DNA damage alone nor protected HepG2 cells from H_2_O_2_ treatment. We could, therefore, suppose that fructosylation highlighted the protective potential of tyrosol.

Our results obtained with HOT are in agreement with Tutino et al. [73], who provided insights into the mechanisms of action of HOT in the context of inhibition of cell proliferation and prevention of oxidative stress in human hepatoma cells. The authors detected an increase in total cellular antioxidant activity after HOT treatment [73]. Our experiments supported this outcome in another manner, as a decreased level of DNA damage induced by H_2_O_2_.

## 3. Materials and Methods

### 3.1. General

The reactions were performed with commercial reagents purchased from Sigma-Aldrich (St. Louis, MO, USA), Acrōs Organics (Geel, Belgium), Alfa Aesar (Karlsruhe, Germany), or Merck (Darmstadt, Germany). Toluene, dichloromethane, and methanol were dried (Na, P_2_O_5_) and distilled before use. Molecular sieves (4 Å) were microwave dried before use. Zinc carbonate basic, 97%, Zn > 58.0% was purchased from Alfa Aesar (Karlsruhe, Germany). All reactions using sensitive reagents were carried out under an argon atmosphere. 

Tyrosol (97%) was purchased from Maybridge (Loughborough, Leicestershire, UK), and hydroxytyrosol was prepared according to [74]. Glycosylations under conventional heating were carried out in a preheated aluminum dry bath block in sealed vials. Microwave-assisted reactions were performed in a Discover CEM-SP microwave synthesizer (300 W maximum magnetron output power) using an external IR temperature measurement (CEM Corporation, Matthews, NC, USA). All microwave reactions were conducted in closed vessels under dynamic reaction conditions and cooled by simultaneous external flow of compressed air. The initial maximum power was set to 300 W. When the reaction temperature was set to 120 °C in the first set of screenings under mentioned conditions, the reaction temperature was reached in approximately 50 s. TLC was performed on aluminum sheets precoated with silica gel 60 F_254_ (Merck, Darmstadt, Germany). Spots were visualized by UV light (λ_max_ = 254 nm) and charred with 5% ethanolic sulfuric acid comprising 1% orcinol. Flash column chromatography was carried out on silica gel 60 (0.040–0.060 mm, Merck, Darmstadt, Germany or 0.035–0.075 mm, Acrōs Organics, Geel, Belgium) using distilled solvents (toluene (T), ethyl acetate (EA), and chloroform, methanol). ^1^H NMR and ^13^C NMR spectra were recorded at 25 °C on 400 MHz Bruker AVANCE III HD equipped with Prodigy CryoProbe (Bruker GmbH, Karlruhe, Germany). Chemical shifts were referenced to either TMS (δ 0.00, CDCl_3_ for ^1^H) or HOD (δ 4.79, CD_3_OD for ^1^H) and to internal CDCl_3_ (δ 77.16, ^13^C) or CD_3_OD (δ 49.00, ^13^C). Chemical shifts (in ppm) and coupling constants (in Hz) were obtained by first-order analysis; assignments were derived from COSY and H/C correlation spectra. The multiplicity of the ^13^C NMR signals concerning the ^1^H–^13^C coupling was determined by the HSQC method. NMR spectra of new compounds are provided in the Supplementary Material. Optical rotations were measured on Perkin–Elmer 241 (PerkinElmer, Waltham, MA, USA) or Jasco P2000 (Jasco Products Company, Oklahoma City, OK, USA) polarimeters at 20 °C. High-resolution mass spectrometry was performed on a Premier Q-TOF mass spectrometer (Waters Corp, Milford, MA, USA) or an Orbitrap Velos PRO spectrometer (Thermo Fisher Scientific, Waltham, MA, USA).

Prepared PEGFs were kept at 4 °C, 0.1 M stock solutions were kept in dimethyl sulfoxide (DMSO) at −20 °C, and dilutions in appropriate reaction mixtures were done freshly just before the experiments. Hydrogen peroxide (H_2_O_2_), ethidium bromide (EtBr), and thiazolyl blue tetrazolium bromide (MTT) were purchased from Sigma-Aldrich Co., St. Louis, MO, USA. All other reagents and chemicals used were of analytical grade. 

### 3.2. Glycosylation Methods

#### 3.2.1. Preparation of Glycofuranosyl Bromides **13**–**15**

To a solution of per-*O*-acylated furanose **9**, **10**, or **11** (5 mmol) dissolved in dry CH_2_Cl_2_ (9 mL) and cooled to 0 °C, trimethylsilyl bromide (3.3 mL, 25 mmol) was added under argon and through the septum. The temperature of the reaction mixture was then allowed to rise to rt and stirred until the starting acetate has completely reacted according to TLC (4–15 h). The reaction mixture was concentrated at a temperature not exceeding 40 °C and used immediately in the next reaction.

#### 3.2.2. General Procedure for 1,2-*trans*-Glycosylation under Conventional Heating Using Basic ZnCO_3_ as a Promoter. Method Δ

Acetylated HOT **12** (57.1 mg, 0.24 mmol) and glycofuranosyl bromide (0.3 mmol, 1.25 equiv) were dissolved in dry toluene (3 mL) by short pre-stirring. After the dissolution of reactants, basic zinc carbonate (57.7 mg, 0.105 mmol, 0.44 equiv), together with powdered 4 Å MS (240 mg), was added to the mixture and the reaction was stirred at 60 °C. After completion of the reaction (monitored by TLC), the reaction mixture was diluted under vigorous stirring with ethyl acetate (30 mL). The heterogeneous mixture was then filtered through Celite 545, and the filter cake was washed with ethyl acetate. The collected filtrate was concentrated to dryness to give a crude product, which was purified by column chromatography on silica gel. The eluent toluene:ethyl acetate (4:1) were used to isolate per-*O*-acylated glycofuranosides **16**–**18**. Yields and anomeric ratios of products are summarized in Table 1.

#### 3.2.3. General Procedure for Microwave-Assisted Glycofuranosylation Using Basic ZnCO_3_ as a Promoter (Method MW)

Acetylated HOT **12** (57.1 mg, 0.24 mmol) and glycofuranosyl bromide (0.3 mmol, 1.25 equiv) were dissolved in dry toluene (3 mL) by short pre-stirring. After the dissolution of reactants, basic zinc carbonate (0.35 or 0.44 equiv), together with powdered 4 Å MS (240 mg), was added to the mixture. The cuvette containing the reaction mixture was placed into a microwave reactor, and the reaction was stirred using a dynamic method at a maximum power 300 W, typically at 60 or 120 °C, for the time indicated in Table 1. After the completion of the reaction (monitored by TLC), the compounds **16**–**18** were isolated from the reaction mixtures as described in the previous methods. Yields and anomeric ratios of the products are summarized in Table 1.

#### 3.2.4. Typical Preparative Procedure for Glycofuranosylation under Conventional Heating Using Basic ZnCO_3_

Glycofuranosyl bromide (5.0 mmol, 1.25 equiv) and acetylated HOT **12** (0.95 g, 4 mmol) were dissolved in dry toluene (33 mL) by short pre-stirring, followed by the addition of 4 Å MS (4 g). The reaction mixture was then heated to 60 °C, followed by the addition of basic zinc carbonate (0.96 g, 1.87 mmol, 0.46 equiv), and the mixture was stirred at 60 °C in an aluminum dry bath block for 50 min. After the completion of the reaction, the mixture was diluted under vigorous stirring with ethyl acetate (15 mL). The suspension was then filtered through Celite 545, and the filter cake was washed with ethyl acetate. The supernatant and washings were combined and evaporated to dryness to give the crude product, which was further purified by chromatography on silica gel (toluene:ethyl acetate 10:1) to afford **16** (1.370 g, 69%), **17** (1.497 g, 67%), or **18** (1.191 g, 60%). 

#### 3.2.5. Characterization Data of Per-O-acylated Glycofuranosides **16**–**18**

2-[3,4-bis(acetoxy)phenyl]ethyl 2,3,5-tri-*O*-acetyl-α-L-arabinofuranoside (**16**). Colorless syrup; R_f_ = 0.43 (T/EA, 1:1, *v*/*v*); [α]_D_^20^ = −74.9° (c = 1.0, CHCl_3_); (Table 1, Entry 1); ^1^H NMR (400 MHz, CDCl_3_), δ 7.11 (dd, *J* = 8.3, 1.8 Hz, 1H, CHPh), 7.08 (d, *J* = 8.2 Hz, 1H, CHPh), 7.06 (d, *J* = 1.6 Hz, 1H, CHPh), 5.05 (d, *J* = 1.6 Hz, 1H, H-2), 5.01 (s, 1H, H-1), 4.95 (dd, *J* = 5.2, 1.6 Hz, 1H, H-3), 4.38 (dd, *J* = 12.0, 3.4 Hz, 1H, H-5a), 4.19 (dd, *J* = 12.0, 5.5 Hz, 1H, H-5b), 4.05 (td, *J* = 5.3, 3.5 Hz, 1H, H-4), 3.93 (dt, *J* = 9.7, 7.0 Hz, 1H, OCH_2_a), 3.72 (dt, *J* = 9.7, 6.3 Hz, 1H, OCH_2_b), 2.89 (t, *J* = 6.6 Hz, 2H, OCH_2_CH_2_), 2.27 (s, 3H, COCH_3_), 2.26 (s, 3H, COCH_3_) 2.09 (s, 6H, 2xCOCH_3_), 2.07 (s, 3H, COCH_3_); ^13^C NMR (101 MHz, CDCl_3_), δ 170.7 (COCH_3_), 170.3 (COCH_3_), 169.8 (COCH_3_), 168.4 (COCH_3_), 168.3 (COCH_3_), 142.0 (CPh), 140.6 (CPh), 137.9 (CPh), 127.2 (CHPh), 123.9 (CHPh), 123.2 (CHPh), 105.6 (C-1), 81.4 (C-2), 80.4 (C-4), 77.1 (C-3), 67.8 (OCH_2_CH_2_), 63.3 (C-5), 35.4 (OCH_2_CH_2_), 20.9 (COCH_3_), 20.9 (COCH_3_), 20.8 (COCH_3_), 20.8 (COCH_3_), 20.7 (COCH_3_); HRMS (APCI): calcd. for C_23_H_28_O_12_ [M + H]^+^ = 497.16535, found 497.16529.

2-[3,4-Bis(acetoxy)phenyl]ethyl 5-*O*-benzoyl-2,3-di-*O*-acetyl-β-D-apiofuranoside (**17**). Colorless syrup; R_f_ = 0.48 (T/EA, 1:1, *v*/*v*); [α]_D_^20^ = −53.9° (c = 1.0, CHCl_3_); (Table 1, Entry 3); ^1^H NMR (400 MHz, CDCl_3_), δ 8.10–8.07 (m, 2H, CHBz), 7.58 (t, *J* = 7.4 Hz, 1H, CHBz), 7.46 (t, *J* = 7.6 Hz, 2H, CHBz), 7.05–7.02 (m, 3H, CHPh), 5.43 (s, 1H, H-2), 5.00 (s, 1H, H-1), 4.83 (d, *J* = 12.2 Hz, 1H, H-5a), 4.74 (d, *J* = 12.2 Hz, 1H, H-5b), 4.17 (s, 2H, H-4), 3.91 (dt, *J* = 9.5, 7.1 Hz, 1H, OCH_2_a), 3.67 (dt, *J* = 9.5, 6.4 Hz, 1H, OCH_2_b), 2.88 (t, *J* = 6.7 Hz, 2H, OCH_2_CH_2_), 2.22 (s, 3H, COCH_3_), 2.21 (s, 3H, COCH_3_), 2.09 (s, 3H, COCH_3_), 2.01 (s, 3H, COCH_3_); ^13^C NMR (101 MHz, CDCl_3_), δ 169.8 (COCH_3_), 169.3 (COCH_3_), 168.4 (COCH_3_), 168.3 (COCH_3_), 166.0 (COPh), 142.0 (CPh), 140.7 (CPh), 133.3 (CHBz), 131.6 (CPh), 130.0 (2xCHBz), 129.8 (CBz), 128.5 (2xCHBz), 127.2 (CHPh), 123.9 (CHPh), 123.3 (CHPh), 105.7 (C-1), 84.1 (C-3), 76.5 (C-2), 72.7 (C-4), 68.4 (OCH_2_CH_2_), 63.9 (C-5), 35.5 (OCH_2_CH_2_), 21.2 (COCH_3_), 20.7 (COCH_3_), 20.7 (COCH_3_), 20.7 (COCH_3_); HRMS (ESI): calcd. for C_28_H_30_O_12_ [M + Na]^+^ = 581.16295, found 581.16339.

2-[3,4-Bis(acetoxy)phenyl]ethyl 2,3,5-tri-*O*-acetyl-β-D-ribofuranoside (**18**). Colorless syrup; R_f_ = 0.39 (T/EA, 1:1, *v/v*); [α]_D_^20^ = −38.8° (c = 1.0, CHCl_3_); (Table 1, Entry 6); ^1^H NMR (400 MHz, CDCl_3_), δ 7.09 (d, *J* = 8.3 Hz, 1H, CHPh), 7.07 (dd, *J* = 8.3, 1.7 Hz, 1H, CHPh), 7.03 (d, *J* = 1.7 Hz, 1H, CHPh), 5.25 (dd, *J* = 6.7, 4.8 Hz, 1H, H-3), 5.21 (dd, *J* = 4.9, 1.0 Hz, 1H, H-2), 4.98 (d, *J* = 1.0 Hz, 1H, H-1), 4.28–4.23 (m, 1H, H-4), 4.23 (dd, *J* = 11.8, 3.7 Hz, 1H, H-5a), 3.95 (dd, *J* = 11.3, 5.8 Hz, 1H), 3.92 (dt, *J* = 9.5, 7.0 Hz, 1H, OCH_2_a), 3.60 (dt, *J* = 9.5, 6.8 Hz, 1H, OCH_2_b), 2.85 (t, *J* = 6.9 Hz, 2H, OCH_2_CH_2_), 2.26 (s, 1H, CH_3_), 2.26 (s, 1H, CH_3_), 2.09 (s, 1H, CH_3_), 2.05 (s, 1H, CH_3_), 2.04 (s, 1H, CH_3_); ^13^C NMR (101 MHz, CDCl_3_), δ 170.7 (COCH_3_), 169.8 (COCH_3_), 169.7 (COCH_3_), 168.4 (COCH_3_), 168.4 (COCH_3_), 142.0 (CPh), 140.7 (CPh), 137.5 (CPh), 127.2 (CHPh), 124.0 (CHPh), 123.3 (CHPh), 105.2 (C-1), 78.7 (C-4), 74.8 (C-2), 71.7 (C-3), 68.4 (OCH_2_), 64.8 (C-5), 35.4 (OCH_2_CH_2_), 20.9 (COCH_3_), 20.7 (COCH_3_), 20.7 (COCH_3_), 20.7 (COCH_3_), 20.6 (COCH_3_); HRMS (ESI): calcd. for C_23_H_28_O_12_ [M + Na]^+^ = 519.14730, found 519.14694.

### 3.3. Deacylation of Per-O-Acylated Glycofuranosides **16**–**18**

Per-*O*-acetylated glycofuranoside (2 mmol) was dissolved in dry MeOH (30 mL) and cooled to 4 °C, and 0.5 M MeONa (3.4 mL) was added dropwise. The mixture was stirred at 4 °C until completion of the reaction was indicated by TLC (EtOAc:MeOH 6:1). The mixture was neutralized to pH 5 with Dowex^®^ 50WX8 (H+ form). The resin was then filtered off, and the filtrate was evaporated to dryness at temperature not higher than 40 °C. The residue was purified by column chromatography on silica gel (EtOAc:MeOH 6:1) and concentrated.

2-(3,4-Dihydroxyphenyl)ethyl α-L-arabinofuranoside (**6**). Yield 44%. Colorless syrup; R_f_ = 0.48 (EA/MeOH, 9:1, *v*/*v*); [α]_D_^20^ = −21.8° (*c* 1.0, CH_3_OH); ^1^H NMR (400 MHz, CD_3_OD), δ 6.72–6.69 (m, 1H, CHPh), 6.68 (d, *J* = 4.1 Hz, 1H, CHPh), 6.57 (dd, *J* = 8.1, 2.1 Hz, 1H, CHPh), 4.90 (d, *J* = 1.7 Hz, 1H, H-1), 3.98 (dd, *J* = 3.7, 1.7 Hz, 1H, H-2), 3.92 (d, *J* = 3.2 Hz, 1H, H-4), 3.89 (dt, *J* = 9.7, 7.2 Hz, 1H, OCH_2_a), 3.84 (dd, *J* = 6.4, 3.7 Hz, 1H, H-3), 3.77 (dd, *J* = 11.8, 3.2 Hz, 1H, H-5a), 3.67 (dd, *J* = 12.1, 5.3 Hz, 1H, H-5b), 3.61 (dt, *J* = 9.7, 7.2 Hz, 1H, OCH_2_b), 2.77 (t, *J* = 7.1 Hz, 2H, OCH_2_CH_2_); ^13^C NMR (101 MHz, CD_3_OD), δ 146.1 (CPh), 144.6 (CPh), 131.8 (CPh), 121.2 (CHPh), 117.0 (CHPh), 116.3 (CHPh), 109.4 (C-1), 85.3 (C-4), 83.5 (C-2), 78.7 (C-3), 70.0 (OCH_2_), 63.0 (C-5), 36.5 (OCH_2_CH_2_); HRMS (ESI): calcd. for C_13_H_18_O_7_ [M + Na]^+^ = 309.09447; found 309.09447.

2-(3,4-Dihydroxyphenyl)ethyl β-D-apiofuranoside (**7**). Yield 36%. Colorless syrup; R_f_ = 0.51 (CHCl_3_/MeOH, 3:1, *v*/*v*); [α]_D_^20^ = −12.9° (*c* 1,0, CH_3_OH). ^1^H NMR (400 MHz, CD_3_OD), δ 6.67 (d, *J* = 8.0 Hz, 2H, CHPh), 6.66 (d, *J* = 2.0 Hz, 1H, CHPh), 6.53 (dd, *J* = 8.0, 2.1 Hz, 1H, CHPh), 4.92 (d, *J* = 2.5 Hz, 1H, H-1), 3.87 (d, *J* = 9.6 Hz, 1H, H-4a), 3.84 (d, *J* = 2.4 Hz, 1H, H-2), 3.80 (dt, *J* = 9.5, 7.2 Hz, 2H, OCH_2_a), 3.73 (d, *J* = 9.7 Hz, 1H, H-4b), 3.57 (dt, *J* = 9.6, 7.0 Hz, 2H, OCH_2_b), 3.55 (d, *J* = 11.5 Hz, 1H, H-5a), 3.51 (d, *J* = 11.5 Hz, 1H, H-5b), 2.71 (t, *J* = 7.1 Hz, 2H, OCH_2_CH_2_); ^13^C NMR (101 MHz, CD_3_OD), δ 146.1 (CPh), 144.6 (CPh), 131.8 (CPh), 121.2 (CHPh), 117.0 (CHPh), 116.3 (CHPh), 110.4 (C-1), 80.4 (C-3), 78.0 (C-2), 74.9 (C-4), 70.5 (OCH_2_), 65.5 (C-5), 36.6 (OCH_2_CH_2_); HRMS (ESI): calcd. for C_13_H_18_O_7_ [M + Na]^+^ = 309.09447; found 309.09457.

2-(3,4-Dihydroxyphenyl)ethyl β-D-ribofuranoside (**8**)**.** Yield 31%. Colorless syrup; R_f_ = 0.50 (CHCl_3_/MeOH, 3:1, *v*/*v*); [α]_D_^20^ = −12.8° (*c* 0.79, CH_3_OH). ^1^H NMR (400 MHz, CD_3_OD) δ 6.67 (d, *J* = 8.5 Hz, 1H, CHPh), 6.65 (d, *J* = 2.0 Hz, 1H, CHPh), 6.53 (dd, *J* = 8.0, 2.1 Hz, 1H, CHPh), 4.85 (1H, H-1, overlapping with CD_3_OD), 4.00 (dd, *J* = 6.9, 4.7 Hz, 1H, H-3), 3.91 (td, *J* = 6.8, 3.4 Hz, 1H, H-4), 3.90–3.83 (m, 2H, H-2 overlapping with OCH_2_a), 3.65 (dd, *J* = 11.7, 3.5 Hz, 1H, H-5a), 3.54 (dt, *J* = 9.5, 6.9 Hz, 1H, OCH_2_b), 3.43 (dd, *J* = 11.7, 6.7 Hz, 1H, H-5b), 2.69 (t, *J* = 7.0 Hz, 2H, OCH_2_CH_2_); ^13^C NMR (101 MHz, CD_3_OD) δ 146.1 (CPh), 144.6 (CPh), 131.8 (CPh), 121.2 (CHPh), 117.1 (CHPh), 116.2 (CHPh), 108.6 (C-1), 84.8 (C-4), 76.3 (C-2), 72.9 (C-3), 70.0, (OCH_2_), 65.2 (C-5), 36.5 (OCH_2_CH_2_); HRMS (ESI): calcd. for C_13_H_18_O_7_ [M + Na]^+^ = 309.09447; found 309.09448.

### 3.4. Biochemical Assays

#### 3.4.1. Antioxidant Assays

Free aglycones **1** and **2** and PEGFs **5a**, **5b**, and **6**–**8** were evaluated for their reducing power according to [75] and [76]. A series of T, HOT, PEGFs, and standard gallic acid (GA) at different concentrations (10–0.01 mM) were prepared and mixed in 1 mL of methanol with 2.5 mL phosphate buffer (0.2 M, pH = 6.6) and 2.5 mL potassium ferricyanide [K_3_Fe(CN)_6_] (1%). The mixtures were incubated at 50 °C for 20 min. Then, trichloroacetic acid (2.5 mL, 10%) was added to each sample and the mixture centrifuged at 900× *g* for 10 min. Finally, 2.5 mL of the upper layer from supernatants was mixed with 2.5 mL of distilled water and 0.5 mL of 0.1% FeCl_3_ and the spectrophotometer GENESYS™ 10 Bio, Spectronic, was used to measure the absorbance at the wavelength of 700 nm for the samples and the standard solutions.

Aglycones and PEGFs were evaluated for their ability to scavenge DPPH (1,1-diphenyl-2-picrylhydrazyl) radicals by the assay modified by [77] and by us [32], as well. In brief, 1 mL of methanolic DPPH solution at the concentration of 0.05 mg/mL was mixed with a series of 50 μL aliquots of various concentrations of T, HOT, and PEGFs (10–0.01 mM). GA was used as a standard in the same concentration range. The test tubes were vigorously shaken and left to incubate in dark at room temperature for 20 min. The absorbance of samples, standard, and control (pure methanol) was measured using a GENESYS™ 10 Bio, Spectronic, spectrophotometer at the wavelength of 517 nm. The percentage of scavenging capacity of T, HOT, and PEGFs was calculated by the formula

Scavenging capacity (%) = 100 × (A*_control_* − A*_sample_*)/A*_control_* (1)

where A*_sample_* is the sample’s absorbance or the absorbance of the standard and A*_control_* is the absorbance of the control‘s reaction (all reagents except the tested compounds). 

#### 3.4.2. DNA Topology Assay

The electrophoretic monitoring of topological changes in the plasmid DNA (pBR322) induced by Fe^2+^ ions was used to detect the DNA-protective/DNA-damaging potential of aglycones and PEGFs as described [78] and [32] in detail. In brief, the reaction mixture (final volume of 10 μL) consisted of plasmid DNA (200 ng in buffer) and either Fe^2+^ alone, or tested aglycones and PEGFs alone, or combinations of tested aglycones and PEGFs with Fe^2+^. Fe^2+^ ions induce in plasmid DNA breaks via free radical formation in a Fenton-like reaction. Analysis of DNA topology was carried out by gel electrophoresis (in 1.5% agarose for 60 min/60 V). The DNA was stained with EtBr (1 mg/mL) and visualized by UV illumination (UV Transilluminator MiniBISPro, DNR Bio Imaging Systems Ltd.). Increase in DNA strand breakage was assayed by measuring the conversion of supercoiled DNA, form I, to relaxed forms II and III. Densitometric quantification of plasmid topology forms (%) was carried out in the program ImageJ 1.53c (Wayne Rasband, National Institutes of Health, Kensington, MD, USA). 

### 3.5. Biological Assays In Vitro

#### 3.5.1. HepG2 Cell Line

Human hepatoma HepG2 cells were used for cytotoxicity assessment and the investigation of potential genotoxic/protective action of aglycones and PEGFs. This cell line represents a useful and suitable tool for the detection of dietary genotoxic mutagens/carcinogens and various xenobiotics that could pose health risks to humans because of its functional drug-metabolizing abilities [79]. HepG2 cells were grown as an adherent culture in Dulbecco‘s Modified Eagle’s Medium supplemented with 10% heat-inactivated fetal bovine serum and antibiotics (penicillin 200 U/mL/streptomycin 100 μg/mL) on plastic surfaces at 37 °C in a humidified atmosphere of 5% CO_2_:95% air. All media and supplements used for the maintenance of cells in culture were purchased from Gibco Life Technologies BRL (Paisley, UK). 

#### 3.5.2. Cell Viability Assessment

For determination of cytotoxicity by MTT assay [71,72], HepG2 cells were seeded into 96-well plates in a density of 2.5 × 10^6^/plate, cultivated for 24 h and treated with T, HOT, and PEGFs (0–2000 µM). After the 48 h treatment, the cells were washed with phosphate buffered saline (PBS; Oxoid Limited, Hampshire, UK) and incubated with a medium containing MTT (1 mg/mL) for further 4 h. Then, the medium was removed and replaced by 200 μL of dimethyl sulfoxide (DMSO; SERVA Electrophoresis GmbH, Heidelberg, Germany). The absorbance was measured at 540 and 690 nm using an xMark™ microplate spectrophotometer (Bio-Rad Laboratories Inc., Berkeley, CA, USA). The viability of HepG2 cells was calculated by the formula

Viability (%) = 100 × A_treated cells_/A_control cells_ (2)

#### 3.5.3. Alkaline Comet Assay (Single-Cell Gel Electrophoresis; SCGE)

For the assessment of DNA-damaging/-protective effects of T, HOT, and PEGFs, the concentration range of 50–200 μM was selected for the 48 h treatment of HepG2 cells (their viability was above 70%), followed by 5 min of incubation with H_2_O_2_. The conventional comet assay procedure was performed in alkaline conditions, as suggested in [80], and modified [32]. In brief, control, aglycones-, and PEGFs-treated HepG2 cells were trypsinized; centrifuged (1200 rpm, 5 min); embedded in 0.75% low-melting-point agarose; and placed onto microscopic slides coated with 1% normal-melting-point agarose. After the solidification of the agarose, the respective part of the slides was treated with H_2_O_2_ (300 μM in PBS on ice in the dark), while the slides not treated with H_2_O_2_ were kept for 5 min in cold PBS. All slides were washed in PBS and placed in a lysis solution consisting of 2.5 M NaCl, 100 mM Na_2_EDTA, 10 mM Tris-HCl (pH = 10.0), and 1% Triton X-100 for 1 h at 4 °C. After lysis, the slides were transferred into an electrophoresis buffer (300 mM NaOH, 1 mM Na_2_EDTA, pH > 13.0) for unwinding (40 min at 4 °C) and subjected to electrophoresis (voltage 75 V/cm, amperage ~300 mA) for 30 min at 4 °C. The slides were then neutralized in 0.4 M Tris-HCl (pH = 7.5), drained, and stained with EtBr (5 μg/mL). 

At least 100 EtBr-stained nucleoids/sample/three slides in one electrophoresis run were scored with a Carl Zeiss AxioImager.Z2 fluorescence microscope using a computerized image analysis Metafer 5 (MetaSystems GmbH, Altlußheim， Germany). As a parameter for the expression of DNA damage, the percentage of DNA in the tail was chosen. 

For statistical analysis of the cell viability results (%) and the mean tail DNA (%), SigmaPlot 12.5 and Prism GraphPad 8.4.3 were used. The normality of the distribution was tested by the Shapiro–Wilk test, and the equality of the data variance was tested by Levene’s test. If normally distributed, differences were tested by unpaired *t*-test. If data were normally distributed but did not pass the equality-of-variance test, differences were tested using unpaired *t*-test with Welch’s correction. If the data were non-normally distributed, a Mann–Whitney U test was used. All tests were two-tailed and performed at the significance level α = 0.05. The *p* < 0.05 was considered statistically significant for all analyses (* *p* < 0.05; ** *p* < 0.01; *** *p* < 0.001; ### *p* < 0.001 for control comparison).

## 4. Conclusions

A series of new hydroxytyrosol 1,2-*trans*-glycofuranosides were prepared by modifying the Koenigs–Knorr conditions, using the environmentally friendly basic zinc carbonate as a promoter. The new glycofuranosides, as well as previously enzymatically prepared T and HOT fructofuranosides, were compared with their aglycones (T and HOT) to determine a possible relationship between their structure, their antioxidant capacity, and their DNA-damaging and DNA-protective potential. The results showed that HOT glycofuranosides and HOT possess significant radical scavenging/antioxidant activities comparable to those of GA. The compounds protected plasmid DNA in a dose-dependent manner in the order HOT = HOTFRU > T > HOTAPI > TYBFRU. For glycofuranosides, the effectiveness of this protection can be explained by the different abilities of their primary hydroxyls to participate in radical stabilization together with *ortho*-diphenols of HOT aglycon. The compounds have no genotoxic effect on human HepG2 cells at the concentrations studied. HOT, HOTFRU, and HOTAPI were the best candidates for protection at the cellular level. However, it should be noted that in the concentration range of 5–500 μM, HOTFRU and HOTRIB were less toxic to cells than HOT, T, and other PEGFs. HOTFRU has been shown to have antioxidant potential comparable to that of HOT at lower toxicity.

## Figures and Tables

**Table 1:** Glycosylation of **12** by glycosyl donors **13–15** using basic zinc carbonate as a promoter in toluene.

Entry	Donor	Heating ^1^ ZnCO_3_ (equiv)	Time (min)	Product	Yield ^3^ (%)	α:β ^4^
1	**13**	Δ, 0.44	80	**16**	70	α only
2	**13**	MW, 0.35	6	**16**	30	75:25
3	**14**	Δ, 0.44	50	**17**	67	β only
4	**14**	MW, 0.44	6	**17**	61	21:79
5	**14**	MW ^2^, 0.44	2.3	**17**	37	12:88
6	**15**	Δ, 0.44	50	**18**	67	β only
7	**15**	MW, 0.44	6	**18**	48	20:80

^1^ Method Δ: The reaction was conducted at 60 °C with 1.25 equiv of a glycosyl donor and 1 equiv of an acceptor with the addition of 4 Å MS (0.08 g per 0.1 mmol of donor). Method MW: The same but the reaction was conducted at 60 °C with 1.25 equiv of a glycosyl donor and 1 equiv of an acceptor. ^2^ The reaction was conducted at 120 °C. ^3^ Isolated yields. ^4^ Anomeric ratios were determined by the integration of appropriate peaks in the ^1^H NMR spectra.

**Table 2:** Antioxidant activity of aglycones **1** and **2** and PEGFs **5a**, **5b**, and **6–8**.

Compound	Concentrations (mM)	Reducing Power ^1^	DPPH Scavenging ^1^
		(Absorbance)	(%)
T (**1**)	10	0.089 ± 0.042	6.710 ± 2.907
	1	0.094 ± 0.012	2.120 ± 1.310
	0.1	0.048 ± 0.027	0.955 ± 0.090
	0.01	0.051 ± 0.043	0.000 ± 0.000
HOT (**2**)	10	nd	82.710 ± 8.200
	1	1.891 ± 0.702	81.137 ± 7.947
	0.1	0.259 ± 0.054	15.763 ± 1.488
	0.01	0.046 ± 0.040	0.000 ± 0.000
TYBFRU (**5a**)	10	0.147 ± 0.038	3.920 ± 1.240
	1	0.099 ± 0.037	1.410 ± 0.040
	0.1	0.051 ± 0.029	0.000 ± 0.000
	0.01	0.051 ± 0.035	0.000 ± 0.000
HOTFRU (**5b**)	10	nd	84.170 ± 14.467
	1	1.788 ± 0.227	78.980 ± 13.150
	0.1	0.285 ± 0.001	14.260 ± 20.167
	0.01	0.116 ± 0.051	5.870 ± 8.301
HOTARA (**6**)	10	nd	81.788 ± 2.861
	1	1.527 ± 0.589	73.895 ± 7.768
	0.1	0.276 ± 0.093	5.600 ± 3.652
	0.01	0.167 ± 0.122	2.143 ± 3.439
HOTAPI (**7**)	10	nd	82.513 ± 3.338
	1	1.883 ± 0.923	74.678 ± 11.425
	0.1	0.274 ± 0.081	5.230 ± 3.775
	0.01	0.124 ± 0.038	1.265 ± 1.189
HOTRIB (**8**)	10	nd	83.210 ± 9.270
	1	2.304 ± 0.045	75.440 ± 19.640
	0.1	0.364 ± 0.045	16.950 ± 10.530
	0.01	0.135 ± 0.057	9.615 ± 13.600
GA	10	nd	91.609 ± 6.011
	1	2.151 ± 0.652	88.454 ± 6.240
	0.1	0.309 ± 0.010	25.720 ± 11.839
	0.01	0.108 ± 0.064	3.088 ± 4.963

^1^ Data represent the means ± standard deviations (SD) of three independent experiments; GA (gallic acid) is shown as a standard in both assays; nd (undetectable): the absorbance value is above the detection limit of a spectrophotometer.

## Data Availability

Not applicable.

## Supplementary Materials

The following are available online, Figures S1–S12: Copies of ^1^H and ^13^C NMR, spectra for new compounds: **16-18, 6-8.**

## Sample Availability

Samples of the compounds are not available from the authors.
