# Supplementary material for: Synthesis of Tyrosol and Hydroxytyrosol Glycofuranosides and Their Biochemical and Biological Activities in Cell-Free and Cellular Assays
Source: Molecules. 2021 Dec 15;26(24):7607. doi: 10.3390/molecules26247607 (PMC8709365; doi:10.3390/molecules26247607)

molecules

MDPI

Supplementary materials

Synthesis of Tyrosol and Hydroxytyrosol Glycofuranosides and Their Biochemical and Biological Activities in Cell-free and Cellular Assays

Peter Kis [1], Eva Horváthová [2], Eliška Gálová [3], Andrea Ševčovičová [3], Veronika Antalová [3], Elena Karnišová Potocká [1], Vladimír Mastihuba [1] and Mária Mastihubová [1,*]

[1] Institute of Chemistry, Slovak Academy of Sciences, 845 38 Bratislava, Slovakia; peter.kis@savba.sk (P.K.); elena.potocka@savba.sk (E.K.P.); vladimir.mastihuba@savba.sk (V.M.)

[2] Cancer Research Institute, Biomedical Research Center, Slovak Academy of Sciences, 845 05 Bratislava, Slovakia; eva.horvathova@savba.sk

[3] Department of Genetics, Faculty of Natural Sciences, Comenius University, 842 15 Bratislava, Slovakia; eliska.galova@uniba.sk (E.G.); andrea.sevcovicova@uniba.sk (A.Š.); veron.antalova@gmail.com (V.A.)

* Correspondence: maria.mastihubova@savba.sk; Tel.: +421-2-59410655

^{1}H and ^{13}C NMR spectra of new synthesized compounds

Figure S1.
^{1}H NMR of 2-[3,4-Bis(acetoxy)phenyl]ethyl 2,3,5-tri-*O*-acetyl-*α*-L-arabinofuranoside

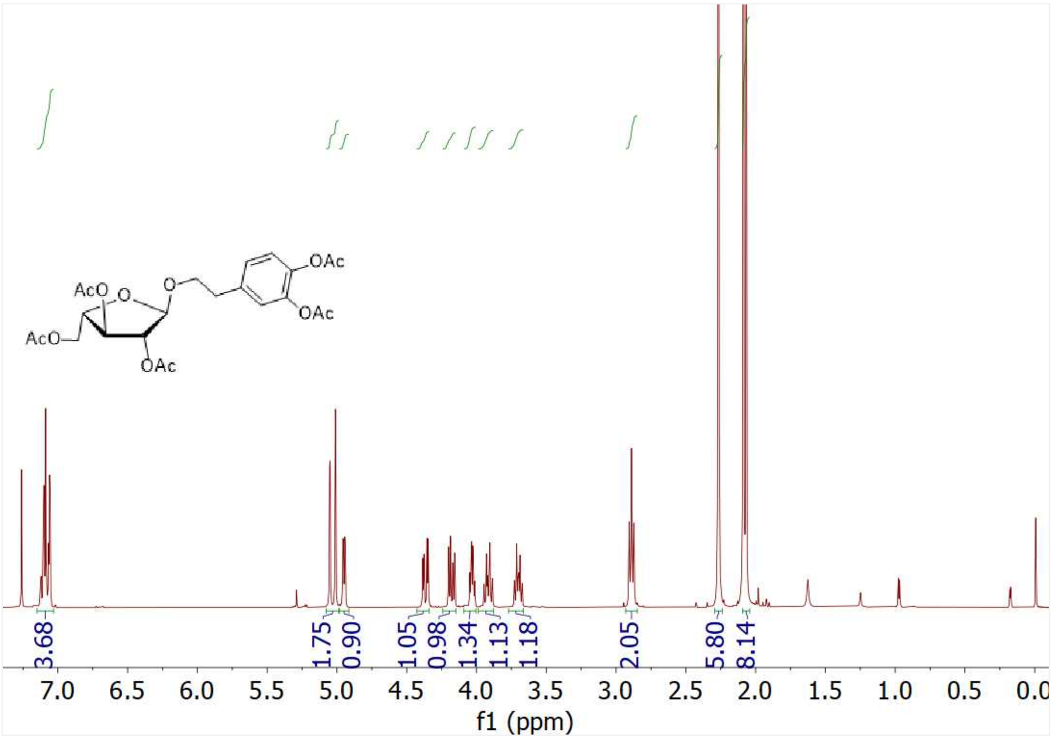

Figure S2.
^{13}C NMR of 2-[3,4-Bis(acetoxy)phenyl]ethyl 2,3,5-tri-*O*-acetyl-*α*-L-arabinofuranoside

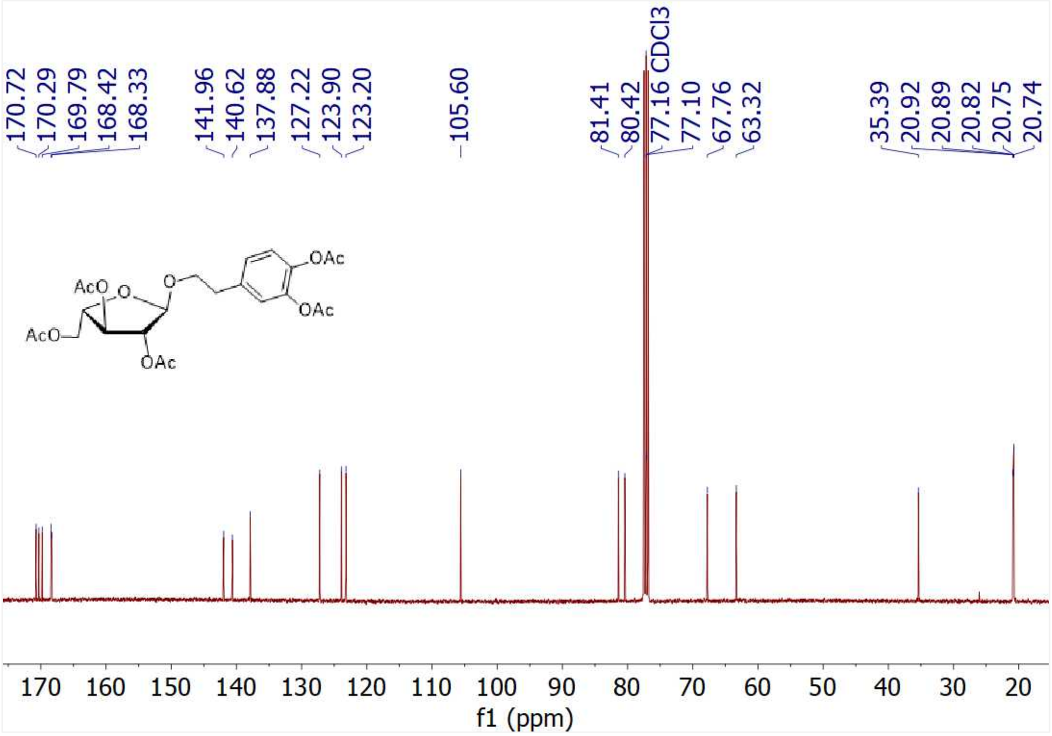

Figure S3.
^{1}H NMR of 2-[3,4-Bis(acetoxy)phenyl]ethyl 5-*O*-benzoyl-2,3-di-*O*-acetyl-β-D-apiofuranoside

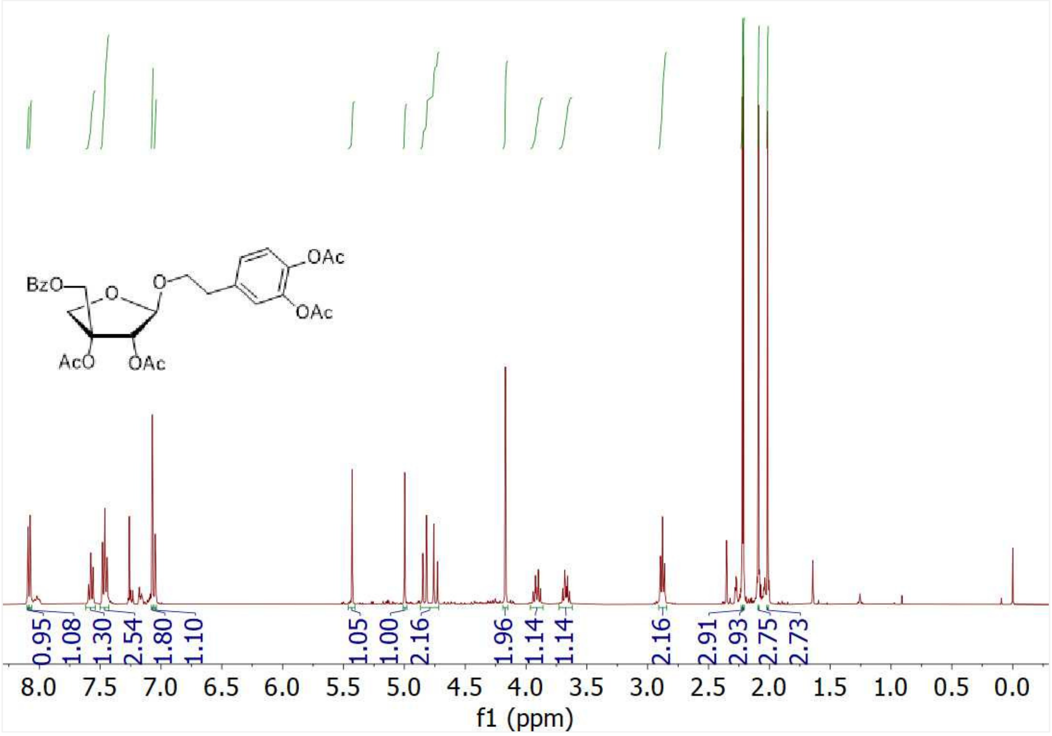

Figure S4.
^{13}C NMR of 2-[3,4-Bis(acetoxy)phenyl]ethyl 5-*O*-benzoyl-2,3-di-*O*-acetyl- *β*-D-apiofuranoside

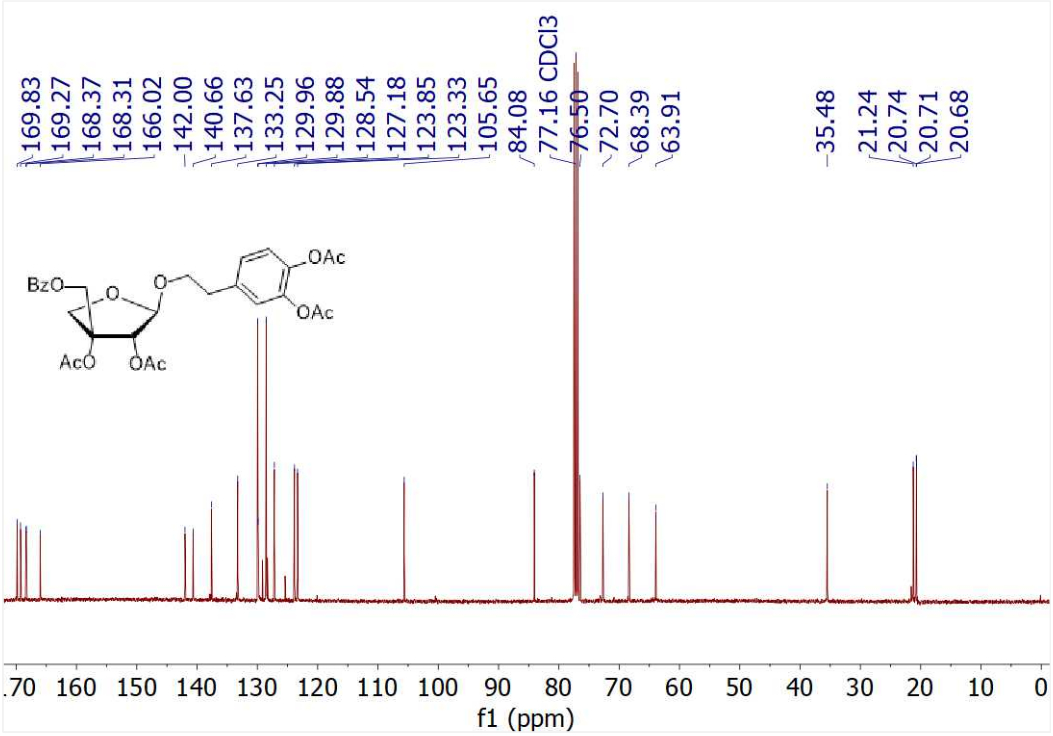

Figure S5.
^{1}H NMR of 2-[3,4-Bis(acetoxy)phenyl]ethyl 2,3,5-tri-*O*-acetyl-*β*-D-ribofuranoside

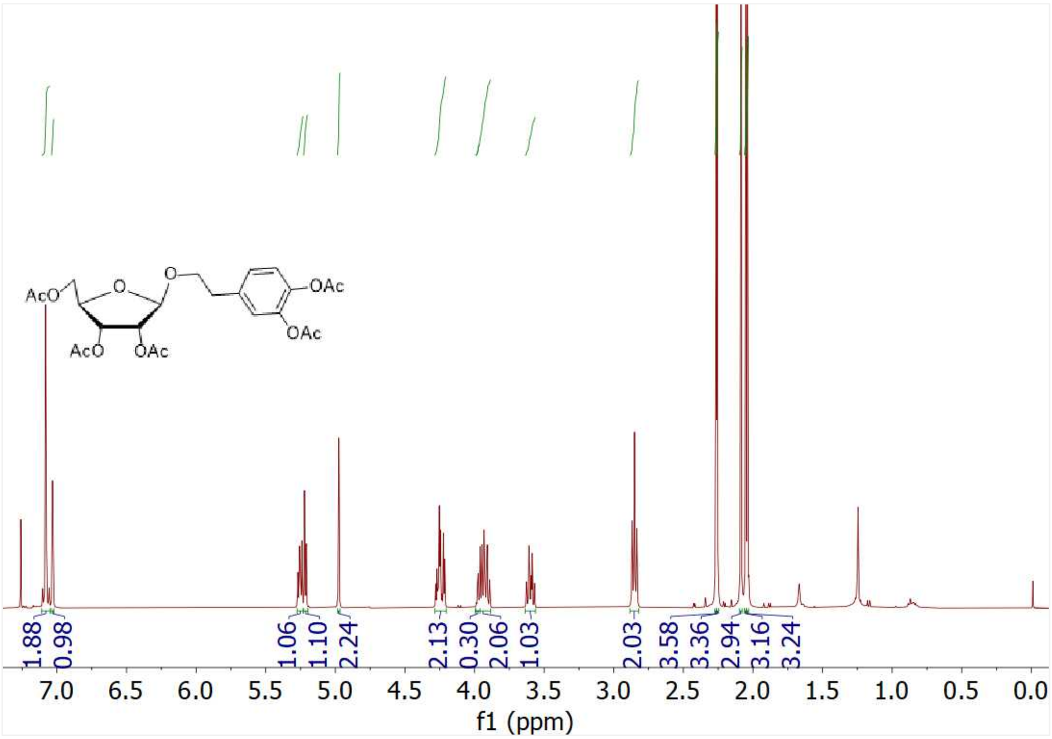

Figure S6.
^{13}C NMR of 2-[3,4-Bis(acetoxy)phenyl]ethyl 2,3,5-tri-*O*-acetyl-*β*-D-ribofuranoside

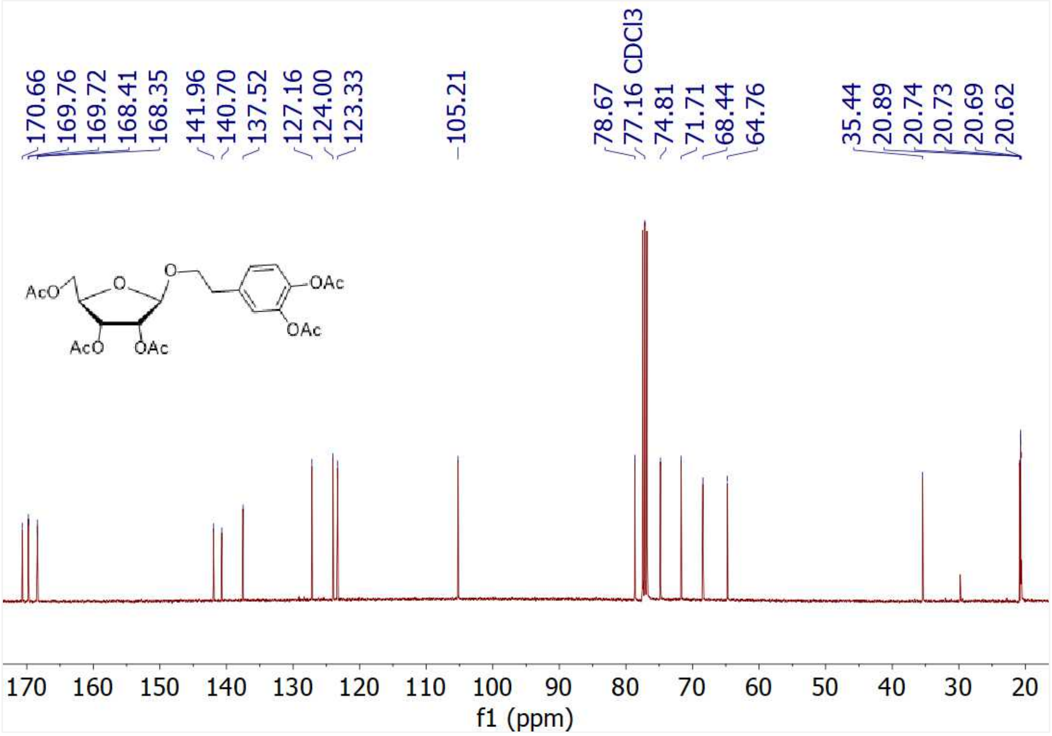

Figure S7.
^{1}H NMR of 2-(3,4-dihydroxyphenyl)ethyl α-L-arabinofuranoside

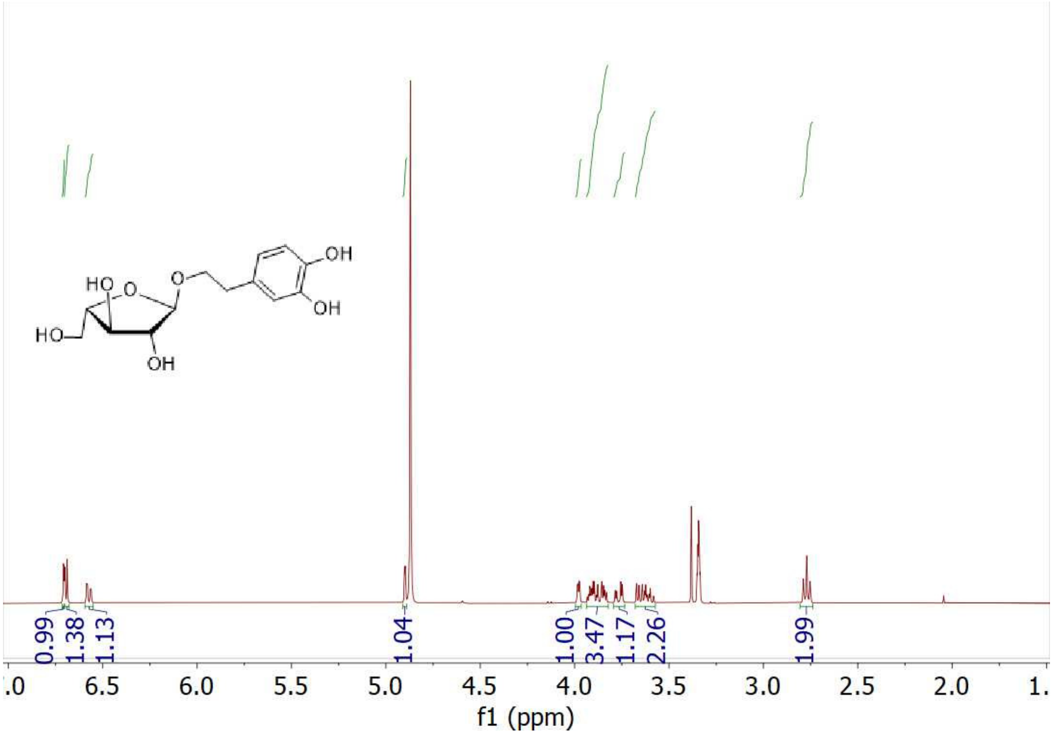

Figure S8.
^{13}C NMR of 2-(3,4-dihydroxyphenyl)ethyl α-L-arabinofuranoside

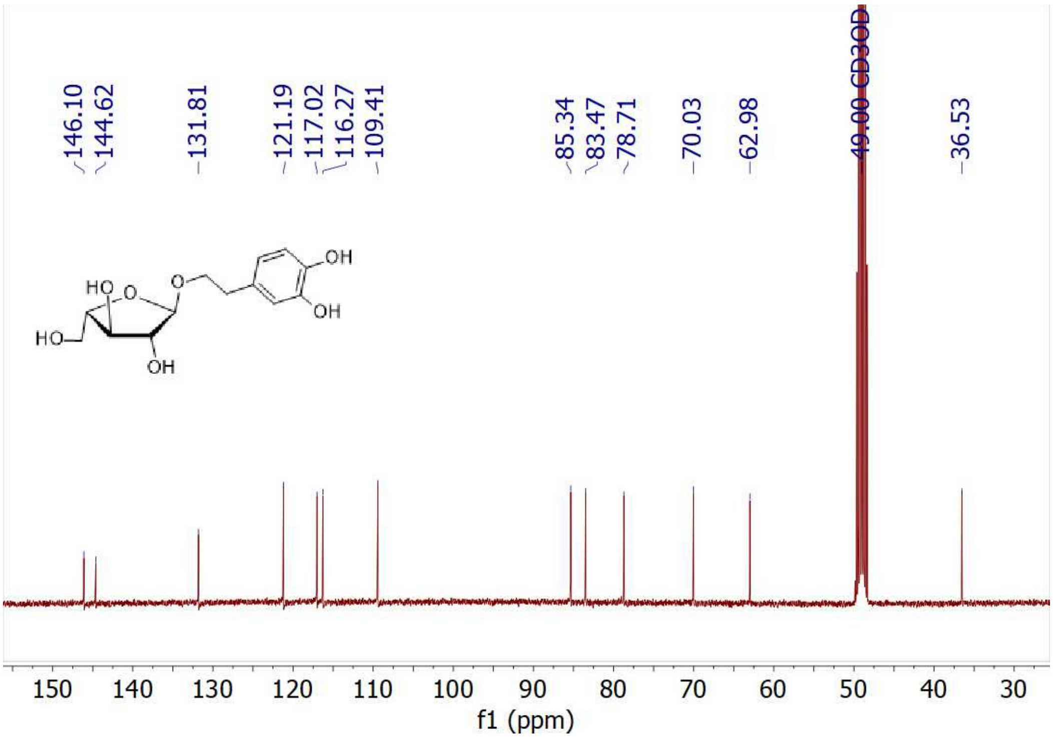

Figure S9.
^{1}H NMR of 2-(3,4-dihydroxyphenyl)ethyl *β*-D-apiofuranoside

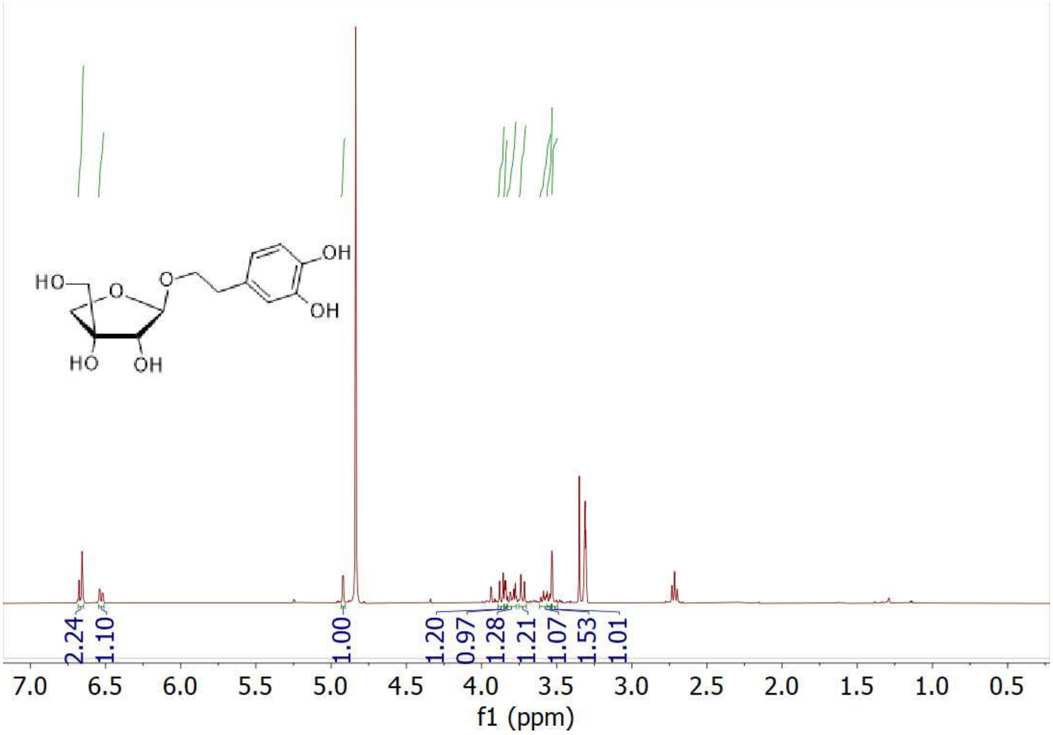

Figure S10.
^{13}C NMR of 2-(3,4-dihydroxyphenyl)ethyl *β*-D-apiofuranoside

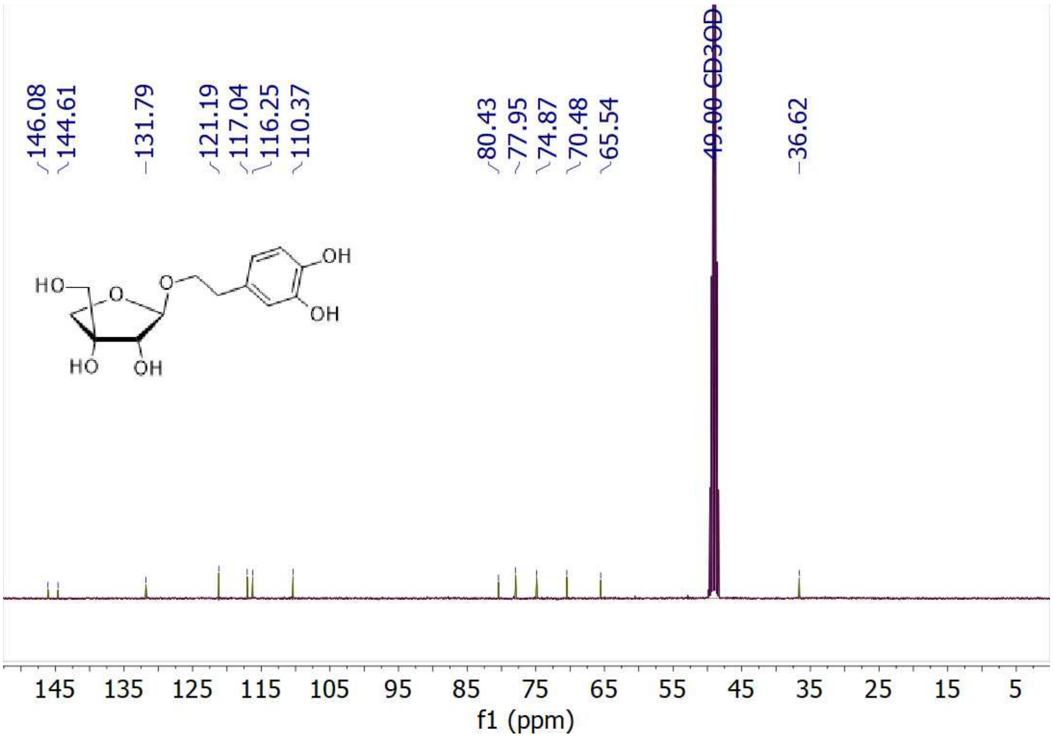

Figure S11.
^{1}H NMR of 2-(3,4-dihydroxyphenyl)ethyl *β*-D-ribofuranoside

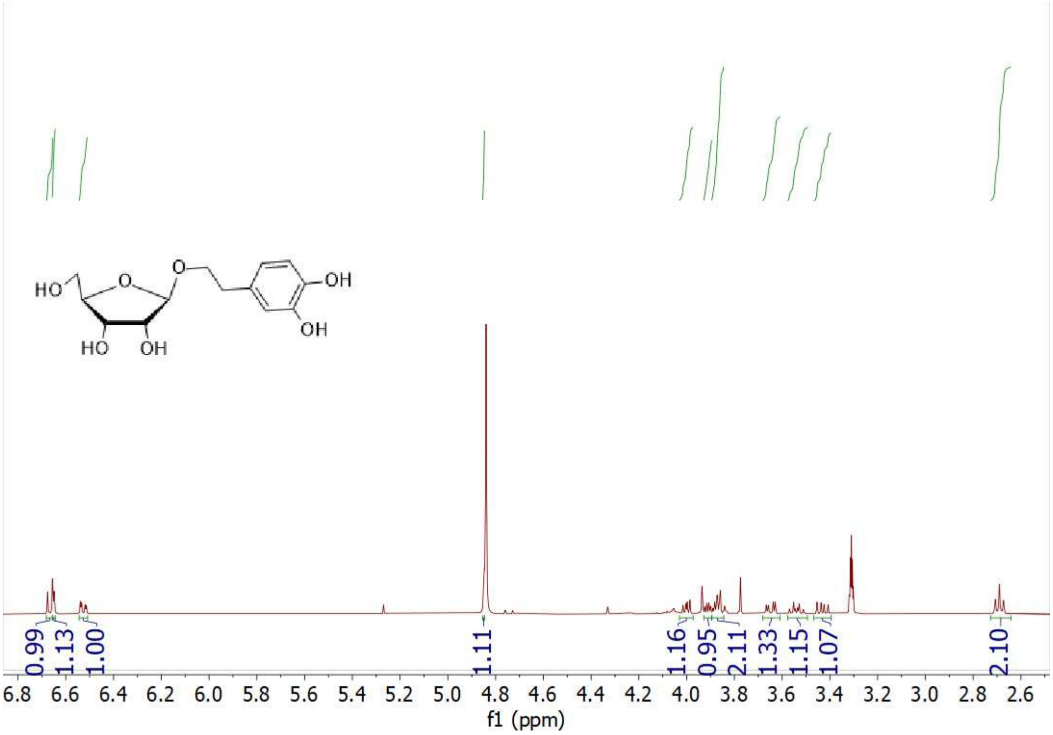

Figure S12.
^{13}C NMR of 2-(3,4-dihydroxyphenyl)ethyl *β*-D-ribofuranoside

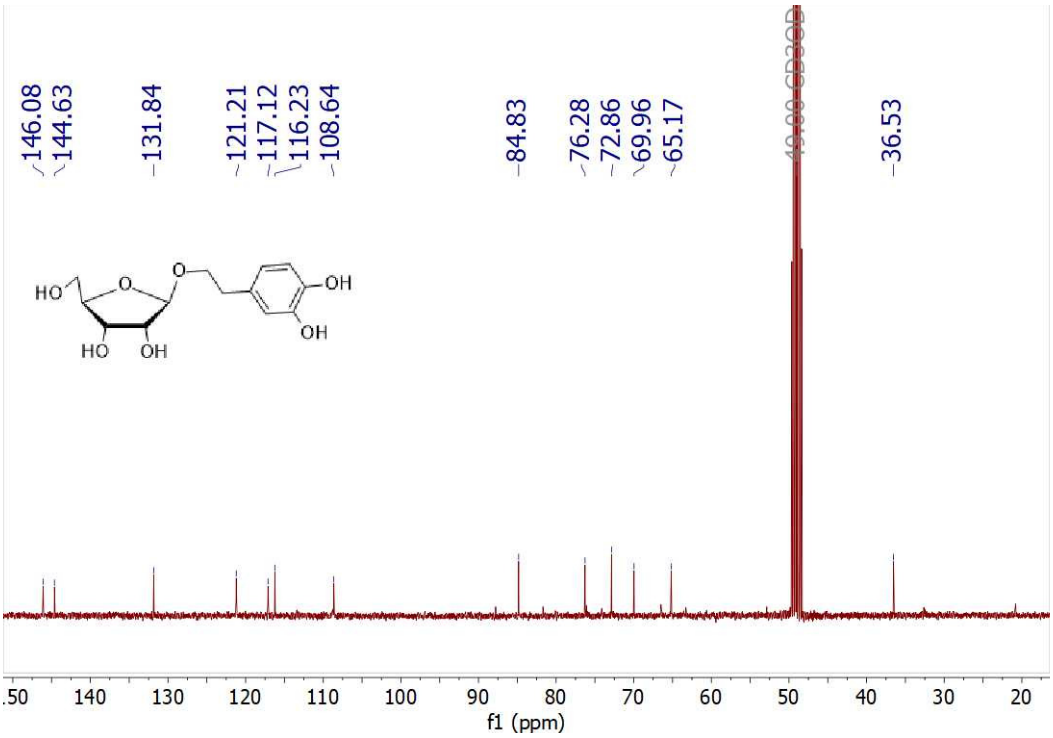

Supplement: Supplementary file 1 [file molecules-26-07607-s001.zip › molecules-1493429-supplementary.pdf]
